# Colostrum immunoglobulins and oxidative capacity may be affected by infant sex and maternal age and parity

**DOI:** 10.3906/sag-1810-66

**Published:** 2019-02-11

**Authors:** Davut Sinan KAPLAN, Cahit BAĞCI, Mustafa ÖRKMEZ, Özge KÖMURCÜ KARUSERCİ, Seyhun SUCU, Hakim ÇELİK, Seyithan TAYSI

**Affiliations:** 1 Department of Physiology, Sakarya University, Sakarya Turkey; 2 Department of Biochemistry, Şehitkamil Public Hospital, Gaziantep Turkey; 3 Department of Gynecology and Obstetrics, Faculty of Medicine, Gaziantep University, Gaziantep Turkey; 4 Department of Gynecology and Obstetrics, Cengiz Gökçek Gynecology and Obstetrics Hospital, Gaziantep Turkey; 5 Department of Physiology, Faculty of Medicine, Harran University, Şanlıurfa Turkey; 6 Department of Biochemistry, Faculty of Medicine, Gaziantep University, Gaziantep Turkey

**Keywords:** Total antioxidant status, total oxidant status, breast milk

## Abstract

**Background/aim:**

The aims of this study were to determine the levels of the total antioxidant status (TAS), the total oxidant status (TOS), the oxidative stress index (OSI), and the concentration of immunoglobulin A (IgA) and M (IgM) in colostrum, and evaluate relationships between these parameters and maternal age, maternal parity, and infant sex.

**Materials and methods:**

The analysis was performed in serum samples of colostrum which were collected from 90 mothers on the first day of lactation between 10:00 and 12:00 AM.

**Results:**

The measurements established that no significant association existed between the TAS level of colostrum and parity, maternal age, or infant sex. However, mothers 18 to 30 years of age had significantly lower colostrum TOS and OSI levels compared with mothers older than 30 years of age. IgA and IgM values of the colostrum of primiparous mothers were significantly higher than those of multiparous mothers, whereas no correlations existed with the age of the mother. Additionally, significantly higher colostrum IgA and IgM values were observed in female infants fed colostrum compared with male infants.

**Conclusion:**

In conclusion, sex-based hormonal changes in mothers during pregnancy may be associated with the different colostral immunoglobulin levels for male and female infants.

## 1. Introduction 

Because optimal growth and development of infants is possible only with breast-feeding, breast milk is considered to be the gold standard for infant nutrition. Breast milk transmits nutrients to the infant, affects biochemical systems, enhances immunity, and eliminates many pathogens.

Increased concentrations of the oxidation products of lipids, proteins, and nucleic acids in bodily fluids indicate oxidative stress. The balance between the oxidation power of reactive oxygen species (ROS) and antioxidant defense mechanisms determines the degree of oxidative stress. The aim of the antioxidant defense system is to protect the organism against the adverse effects of ROSs (1). Infants are exposed to increased oxidative stress due to increased ROS production during delivery and a decrease in antioxidative components, such as vitamin E or glutathione peroxidase (2). Immunoglobulin A (IgA) is the principal immunoglobulin in colostrum and milk. IgA concentrations decline by the fourth week postpartum. However, significant levels of IgA are maintained in milk during the first year of lactation and in the second year of life, milk contains considerable amounts of IgA even if a baby is partially breast-fed (3). IgA levels may vary according to the personal characteristics of the mother and the gestational age of the baby. More IgA and immune factors are found in the colostrum and the milk of mothers who deliver preterm ( <37 weeks) compared to a longer birth term (37–42 weeks) (4). The IgM concentration in colostrum is low and decreases progressively during lactation because IgM is produced by the neonate soon after birth in response to infection (5). Compared to IgA concentrations, IgM concentrations exhibit less variability associated with the personal characteristics of the mother and the gestational age of the baby. No changes in IgM concentrations were observed in the colostrum and milk of mothers who gave birth at preterm compared to those of mothers who gave birth at full term (5,6). Similar to IgA, IgM levels were higher in primiparous mothers than in multiparous mothers (7). 

The purpose of the current study was to (1) investigate the levels of the total antioxidant status (TAS), the total oxidant status (TOS), the oxidative stress index (OSI), and the concentration of IgA and IgM in the colostrum, and (2) evaluate relationships between these parameters and maternal age, maternal parity, and infant sex. 

## 2. Materials and methods

### 2.1. Subjects 

This study was performed according to the rules of the ethics committee of Gaziantep University (Gaziantep, Turkey) and with informed consent from the mothers. Inclusion criteria for mothers were spontaneous vaginal delivery (SVD) at term, absence of premature or postmature birth, absence of any complications of pregnancy, delivery, and puerperium (particularly hypertension, eclampsia, infection, dystocia, or agalactia), and absence of plastic surgery in mammary. Inclusion criteria for the neonates comprised a birth weight of 2500–4000 g, absence of fetal distress, birth asphyxia, sepsis, major congenital abnormalities, or other significant diseases.

### 2.2. Sample collection and preparation

Colostrum samples were obtained from mothers on the first day of lactation between 10:00 and 12:00 AM from January to March 2013 using a commercial breast pump. Samples were frozen immediately and stored at −80 °C until analysis. Collection dates and times were recorded as were the demographic and anthropometrical characteristics of the infants and the mothers (Table 1). The previous suckling occurred approximately 2 h prior to sample collection. 

**Table 1:** Maternal and infant characteristics.

Characteristics	N (%)	Mean ± SD(min–max)
Maternal age		28.11 ± 6.8
18–30	61 (67.8)	
>30	29 (32.2)	
Parity		3.13 ± 1.9
Primiparous	18 (20)	
Multiparous	72 (80)	
Sex		
Female	39 (43.3)	
Male	51 (56.7)	
Birth weight (g)	90 (100)	3215.92 ± 387(2500–4000)
Birth height (cm)	90 (100)	49.78 ± 0.7(48–52)
Birth week	90 (100)	39.24 ± 0.9(37–40)
Type of delivery	90 (100)	SVD

Colostrum samples were prepared using a double centrifugal process. Samples were allowed to reach ambient temperature until dissolved. After thawing, colostrum samples were centrifuged for 10 min at 680 × *g* at 4 °C, after which the lipid layer and cellular elements were removed. The aqueous fraction was centrifuged again for 30 min at 10,000 × *g* at 4 °C, and the lipid layer and cellular elements were removed. Clear, ready-to-use serum fractions were separated into 1.5 mL sterile tubes for measurements (8). 

### 2.3. Measurement of colostrum TAS, TOS, and OSI levels

The TAS, TOS, and OSI levels of the serum were measured using an automated analyzer (Tokyo Boeki, Prestige 24, Tokyo, Japan) with a commercially available measurement kit for TAS (Rel Assay Diagnostics, Gaziantep, Turkey) (9) and TOS (Rel Assay Diagnostics, Gaziantep, Turkey) developed by Erel (10). The OSI index was expressed as a percentage ratio of the TOS level to the TAS level. For calculation, the units of the TAS concentration were changed to mmol/L, and the OSI value was calculated according to the following formula: OSI (arbitrary unit) = TOS (μmol H2O2 equiv/L) / TAS (mmol Trolox equiv/L) (11).

### 2.4. Determination of colostrum IgA and IgM concentrations

Serum IgA and IgM levels were detected by nephelometry (BN ProSpec, Siemens Healthcare Diagnostics, Marburg, Germany) using commercially available kits (Siemens N Antiserum to Human IgA, Siemens N Antiserum to Human IgM) according to the manufacturer’s instructions. 

#### 2.4.1. Statistical analysis

Statistical analyses were performed using the Statistical Package for Social Science (SPSS) Version 22.0. Multiple linear regression models were built for comparison of adjusted means with the colostrum samples and also for statistical comparisons between all group measurements. Descriptive statistics for numerical variables were expressed as the mean ± standard deviation (SD); P-values less than 0.05 were considered significant.

## 3. Results

Maternal and infant characteristics are listed in Table 1. The levels of TAS, TOS, and OSI values determined in the colostrum of mothers are reported in three primary categories, i.e. maternal age, maternal parity, and infant sex, on the first day of lactation (Table 2).

**Table 2:** Levels of TAS, TOS, and OSI.

Groups	TAS (mmol Trolox equiv/L)	P	Regression coefficients	TOS (μmolH2O2 Equiv/L)	P	Regression coefficients	OSI	P	Regression coefficients
Sex			B		B			B	
Female	1.85 ± 0.2	0.90	0.006	2.81 ± 1.6	0.51	−0.21	0.15 ± 0.08	0.46	−0.01
Male	1.84 ± 0.2			2.79 ± 1.3			0.15 ± 0.06		
Maternal age									
18–30	1.84 ± 0.2	0.51	0.033	2.57 ± 1.3	0.04*	0.73	0.14 ± 0.06	0.04*	0.03
>30	1.86 ± 0.3			3.28 ± 1.4			0.17 ± 0.07		
Parity									
Primiparous	1.90 ± 0.2	0.17	−0.08	2.76 ± 1.3	0.48	−0.28	0.14 ± 0.05	0.73	−0.07
Multiparous	1.83 ± 0.2			2.81 ± 1.4			0.15 ± 0.07		
General mean	1.84 ± 0.2	-		2.80 ± 1.4	-		0.15 ± 0.07	-	

Data are presented as mean ± SD, and P-value from multiple linear regression analysis. * P < 0.05.

As anticipated, no significant differences were found in TAS levels of colostrum across all three groups. The mean TOS levels were significantly reduced in the colostrum of mothers aged 18–30 years (2.57 ± 1.3) compared with mothers aged >30 years (3.28 ± 1.4) (P = 0.038). No significant differences were determined between primiparous and multiparous mothers. Additionally, no differences in the TOS levels in colostrum were observed between male and female infants. The OSI provided an integrative perspective for TAS and TOS levels in the colostrum. In the current study, the OSI ratios in the colostrum of mothers were observed to be higher after age 30, when compared with the colostrum of mothers aged 18–30 (P = 0.042). No significant differences were found between primiparous and multiparous mothers. No differences were determined for the colostrum OSI between male and female infants.

IgA and IgM concentrations (Table 3) were significantly higher in the colostrum of mothers of female infants compared to mothers of male infants and higher in the colostrum of primiparous compared with multiparous mothers. No significant differences existed between maternal age and immunoglobulin concentrations.

**Table 3:** Concentrations of IgA and IgM.

Groups	IgA (g/L)	P	Regressioncoefficients	IgM (g/L)	P	Regressioncoefficients
Sex			B			B
Female	33.75 ± 15.0	0.01*	8.80	2.12 ± 1.4	0.01*	0.83
Male	25.82 ± 14.2			1.45 ± 0.9		
Maternal age						
18–30	29.16 ± 15.2	0.62	1.76	1.80 ±1.2	0.86	0.06
>30	29.75 ± 14.9			1.57 ±1.1		
Parity						
Primiparous	35.95 ± 15.7	0.01*	−10.5	2.73 ± 1.3	0.001*	−1.53
Multiparous	27.33 ± 14.3			1.54 ± 1.0		
General mean	29.37 ± 15.0			1.74 ± 1.2		

Data are presented as mean ± SD, and P-value from multiple linear regression analysis. * P < 0.05.

## 4. Discussion

Breast milk and colostrum contain many antioxidant constituents, including albumin, bilirubin, cysteine, uric acid, glutathione, coenzyme Q10, lactoferrin, and vitamins A, C, and E (12–14). In addition, breast milk contains several times greater concentrations of antioxidant enzymes catalase, superoxide dismutase, and glutathione peroxidase than serum (15,16). Prior studies have noted that breast-fed children may have higher albumin, uric acid, vitamin C, and TAS levels than formula-fed children do, even when formula milk contained much higher concentrations of antioxidants and vitamins in vitro than fresh breast milk did (12,17). Reports have shown that TAS was higher in the colostrum than in transitional and mature milk (18) and that the TAS levels of colostrum was not affected by storage at −80 °C (19). Each of these studies demonstrated the importance of colostrum and mature milk consumption as an antioxidant source to decrease the risk of disease due to the occurrence of an imbalance between antioxidant status and oxidative stress.

In this study, the importance of TAS in colostrum samples on the first day of lactation was assessed. No significant effects of maternal age, maternal parity, or infant sex on colostrum TAS levels were observed. This study demonstrated that TAS levels were constant and did not change according to maternal or infant characteristics. Consequently, colostrum is a constant source of antioxidants that protect newborns postnatally from higher oxygen levels than encountered prenatally. 

Another finding was that TOS levels in the colostrum were approximately 5 times less than in the blood serum of healthy people (20,21). Both the high persistence of TAS levels in the colostrum relative to blood serum (20) and the up to 5 times less levels of TOS in the colostrum may indicate that antioxidants and oxidants are selectively passed to the colostrum, thereby providing increased evidence of colostrum as important to life support. Older mothers reported significantly higher TOS levels than younger mothers (Table 3). In addition, this study reported no significant difference between maternal parity and infant sex with TOS levels of colostrum compared to the TAS levels. Although the colostrum TOS value exhibited concentrations approximately five times less than in blood serum, an increased maternal age caused increased transport of oxidants to the colostrum. Therefore, it is possible to hypothesize that maternal age is associated with colostrum quality.

The OSI is an important parameter that reveals the general oxidant and antioxidant balance of colostrum on the first day of lactation because it is obtained from proportioning the TAS and TOS levels. The OSI values obtained in this study were significantly associated with maternal age.

Colostrum contains more immunoglobulin than mature milk and its concentration changes throughout lactation (22). In contrast to our research, studies in which milk samples were collected over longer lactation periods, i.e. 1–3 or 1–5 days, report IgA concentrations in the colostrum that differ from those reported in this study and among each other, such as 10 g/L (23), 12 g/L (24), 5.61 g/L (6), 6.5 g/L (4), 2.25 g/L (5), and 17.4 g/L (25). However, Hennart et al. reported dramatic differences in the concentration of IgA between the first day and second day of lactation. On the second day of lactation, colostrum IgA concentrations were decreased by nearly two-fold (26). Comparable studies reported that total protein concentrations were also decreased in colostrum by approximately two-fold between the first and third days of lactation (27). A number of authors demonstrated that the colostrum of primiparous mothers contained higher IgA concentrations compared to multiparous mothers (29,30), while others reported no difference in the colostrum and mature milk (6,26). In the present study, colostrum IgA concentrations were determined to be significantly different between primiparous mothers and multiparous mothers, 35.95 g/L and 27.33 g/L, respectively. This unique spectrum of antibody specificity is achieved by the “homing” of B lymphocytes, synthesized in the mother’s gastrointestinal tract and transferred to her mammary glands. The antibody composition of breast milk compensates partly for the deficiency of antibodies directed against enteric antigens in placentally transferred IgG (31). In prolonged births in primiparous mothers, fetuses can be exposed to enteric pathogens for a long time, so they need more immunoglobulin. This study demonstrated that no significant difference existed between IgA concentrations and maternal age, and was similar to that published previously for colostrum (6,32). A sufficient amount of IgA is vital for a newborn regardless of the age of the mother.

In conclusion, sex-based hormonal changes in mothers during pregnancy may be associated with the different colostral immunoglobulin levels for male and female infants. The results of this study indicate that IgA levels were significantly higher in colostrum consumed by female infants. This study provides an exciting opportunity to advance our knowledge of sex-based changes in breast milk. Important in the first days of life, the presence of higher amounts of immunoglobulins, which are found in female infants compared to male infants, could suggest better protection against various diseases. In general, studies comparing infant sex and disease, such as middle ear infections (33), wheezing (34), necrotizing enterocolitis (35), sudden infant death syndrome (36), growth retardation (37), sepsis (38), and deaths due to asphyxia (39), remarkably report the occurrence of disease significantly more often in male infants. Further research should be undertaken to investigate the relationship between colostral immunoglobulin levels and newborn’s disease.

Newborns have IgM production capacity to protect against infection. For this reason, the rate of IgM tends to fall gradually throughout breast-feeding (5). Similar to IgA concentrations, the colostrum of primiparous mothers contained higher IgM concentrations compared to multiparous mothers (7). Although it is not yet clear exactly how the number of births to an individual mother influences the composition of the colostrum, previous studies reported an effect on certain hormones, such as prolactin (40). This study appears to be consistent with other research that found no difference between maternal age and the amount of IgM in the colostrum (6). The present study determined that, similar to IgA, IgM levels were significantly higher in the colostrum consumed by female infants. Male sex was considered immunologically disadvantageous compared to female infants.
